# Clinical Outcomes among Asymptomatic or Mildly Symptomatic COVID-19 Patients in an Isolation Facility in Chennai, India

**DOI:** 10.4269/ajtmh.20-1096

**Published:** 2020-11-17

**Authors:** Narayanasamy Krishnasamy, Murugan Natarajan, Arunkumar Ramachandran, Jeromie Wesley Vivian Thangaraj, Theranirajan Etherajan, Jayanthi Rengarajan, Meenakshi Shanmugasundaram, Anuradha Kandasamy, Ramesh Ramamoorthy, Arul Velusamy, Naganath Babu Obla Lakshmanamoorthy, Prabhuraman Kanagaraman, Mohammed Iliyas Rahamathula, Geetha Devadas, Babu Peter Sathyanathan, Poonguzhali Rajaji, Karthick Rajendran, Priyadarshini Panneerselvam, Muthukumaran Rajaram, Mohan Panjacharam

**Affiliations:** 1 Madras Medical College and Rajiv Gandhi Government General Hospital, Chennai, India;; 2 Indian Council of Medical Research (ICMR), National Institute of Epidemiology (NIE), Chennai, India;; 3 Department of Psychiatry, Salem Medical College, Salem, India

## Abstract

Globally, India has reported the third highest number of COVID-19 cases. Chennai, the capital of Tamil Nadu state, witnessed a huge surge in COVID-19 cases, resulting in the establishment of isolation facilities named COVID Care Center (CCC). In our study, we describe the demographic, epidemiological, and clinical characteristics; clinical progression; and outcome of 1,263 asymptomatic/mildly symptomatic COVID-19 patients isolated in one such CCC between May 4, 2020 and June 4, 2020. Around 10.5% of the patients progressed to moderate/severe illness, requiring referral for tertiary care, and three died. Nearly half (49.5%) of the patients were symptomatic at the time of admission, 2.2% of the patients developed symptoms post-testing, and 48.5% patients remained asymptomatic during the entire course of illness. Most common presenting symptoms were fever (69.9%) and cough (29.6%), followed by generalized body pain, breathlessness, and loss of smell and taste. On multivariate analysis, we identified that symptomatic patients with comorbidities and higher neutrophil–lymphocyte ratio (NLR) were more likely to progress to severe illness warranting referral for tertiary care. COVID Care Center ensured case isolation and monitoring of asymptomatic/mildly symptomatic patients, thereby providing hospital beds for sick patients. COVID Care Center isolation facilities are safe alternatives for medical institutions to isolate and monitor COVID-19 patients. Older symptomatic patients with comorbidities and a high NLR admitted in an isolation facility must be frequently monitored for prompt identification of clinical progression and referral to higher center for advanced medical care.

## INTRODUCTION

In the last week of December 2019, China witnessed an emergence of a new respiratory disease named COVID-19, caused by SARS-CoV-2. SARS-CoV-2 infection had spread worldwide, affecting more than 218 countries with 42.9 million cases including 1.1 million deaths reported as on October 26, 2020.^1^ India reported its first laboratory-confirmed case of SARS-CoV-2 from the state of Kerala in the later part of January 2020.^2^ As on October 26, 2020, India ranks second in terms of burden of COVID-19 cases globally, with 7.9 million cases including 119,014 deaths. Even though India has the highest number of COVID-19 cases, the fatality rate due to COVID-19 infection is lower than the global fatality rate.^1,2^ Within India, Maharashtra, Tamil Nadu, Andhra Pradesh, Karnataka, Kerala, Uttar Pradesh, and Delhi states have contributed to more than 65% of cases. Densely populated urban cities have been more affected than rural areas.^3,4^ There is also heterogeneity in the mortality rates among states. Tamil Nadu state with South Asian ethnic population has reported around 711,713 cases and 10,956 deaths, with a mortality rate of 1.5%. Chennai city, the capital of Tamil Nadu, contributed nearly half of COVID-19 cases reported in the state of Tamil Nadu.^5^ Chennai saw a surge in cases from the last week of April 2020. The government of Tamil Nadu and Greater Chennai Corporation used test, isolate, and trace strategy to contain the spread of virus. During the initial stage of the outbreak, all patients were admitted in hospital/medical institutions. In view of increasing number of cases, the government of Tamil Nadu established makeshift temporary medical care isolation centers named as COVID Care Center (CCC) in Chennai. COVID Care Centers were established by converting the existing exhibition centers, trade centers, and educational institutions into isolation centers, providing medical care, clinical monitoring, food, and shelter. Asymptomatic and mildly symptomatic patients were admitted in these centers. Establishment of CCC made hospital beds available for moderate and severely ill patients in hospital/medical institutions. In this article, we describe the demographic, epidemiological, and clinical characteristics; clinical progression; and outcome details of 1,263 COVID-19 patients in one such CCC isolation facility in Chennai.

## METHODS

Patients tested positive for SARS-CoV-2 by real-time reverse transcription PCR (rRT-PCR) assays of the upper respiratory tract (nasal and pharyngeal) and classified as asymptomatic and mildly symptomatic were isolated in the CCC. COVID-19 patients who initially got admitted to the state-run tertiary hospitals after clinical evaluation if found to be asymptomatic and mildly symptomatic were also referred to CCC. Greater Corporation of Chennai which had coordinated the field activity of testing and tracing of contacts of cases also referred asymptomatic or mildly symptomatic persons directly or through screening centers to the CCC. The study was carried out in Nandambakkam CCC, the first such facility to be established in Chennai city. The CCC can accommodate 500 patients at a time. Physicians, nursing staff, and other hospital staff were deployed from other state-run medical institutions/hospitals to the CCC for close clinical monitoring of the isolated patients.

All patients admitted to the CCC had their body temperature checked, blood pressure measured by digital sphygmomanometer, and oxygen saturation (SpO_2_) and pulse rate recorded with a finger pulse oximeter. Basic hematological and radiological investigations including complete blood count and a chest skiagram were performed. Patients were monitored for vital parameters thrice a day. For discharge or home quarantine, the criteria set forth by the Ministry of Health and Family Welfare were followed.^2,5^ Patients with significant symptoms or abnormal vitals or abnormal radiologic findings were immediately shifted to a tertiary care hospital in ambulance.

### Data collection.

All patients who tested positive for SARS-CoV-2 admitted in the CCC were included in the study. Information regarding the patients’ demographic details, contact and exposure history, clinical symptoms and signs, clinical progression and outcomes, date of specimen collection, and comorbidities was captured in a case record form. The clinical details of the patients referred to tertiary care centers were obtained from the case sheets of the patients or from the medical records department. Apart from case record forms and case sheets, patients were telephonically contacted to know about their current status or clinical outcome and also to collect information which was missed in the records. Patients who could not be contacted despite repeated efforts were excluded from the outcome’s analysis. The study was approved by the Institutional Ethics Committee of Madras Medical College, Chennai (IEC No: 04042020). Oral consent was obtained from all the patients. For patients younger than 12 years of age, consent was obtained from their parents or their closest relative admitted along with them.

### Statistical analysis.

Categorical data were presented as percentages. Continuous variables were presented as median and interquartile range (IQR) values. Crude odds ratios (ORs) and 95% CIs associated with different exposures were calculated. Variables with *P*-value ≤ 0.05 in univariate analysis were included in the final unconditional multiple logistic regression model. All statistical analyses were conducted using IBM SPSS Statistics for Windows, version 22.0 (IBM Corp., Armonk, NY).

## RESULTS

**Figure 1.**
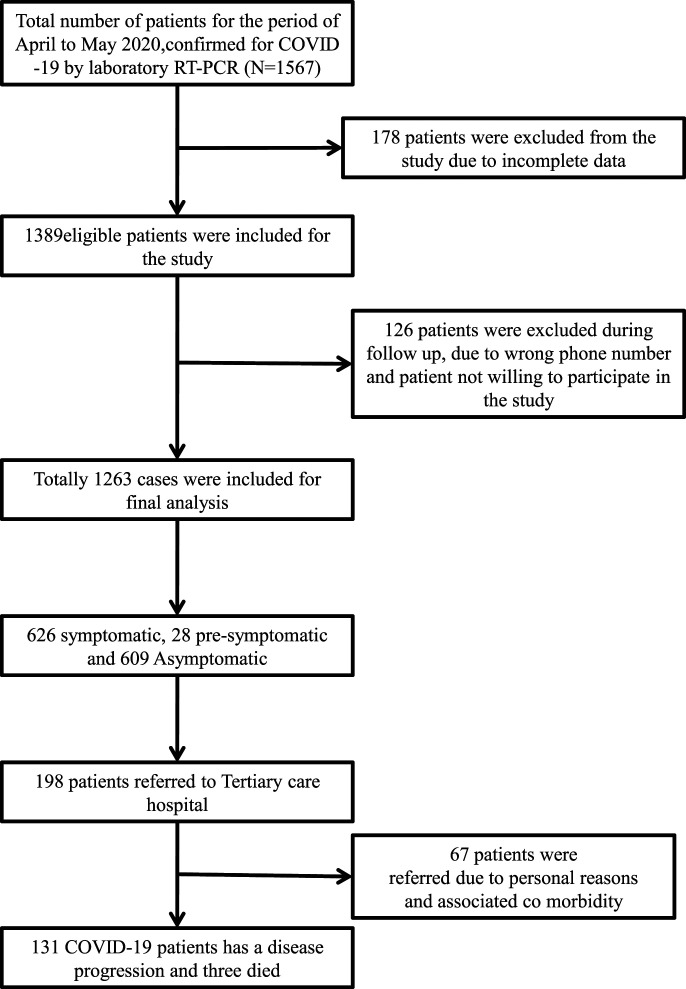
Flowchart showing clinical outcomes of asymptomatic and mildly symptomatic patients isolated in a COVID care center, Chennai, Tamil Nadu, India.

A total of 1,567 laboratory-confirmed COVID-19 patients by RT-PCR were admitted between May 4, 2020 and June 4, 2020 in the study CCC. Of these, 178 patients were excluded from analysis because of incomplete data, another 126 patients could not be telephonically followed up or did not give consent for the study, and 1,263 patients’ records were available for analysis. Of 1,263 patients, 131 (10.5%) progressed to moderate/severe illness, and three died (Figure 1). Rest were discharged alive from the CCC. The median age of COVID-19 patients isolated at the CCC was 35 years (IQR: 25–45 years), and around two-thirds (66.3%) were male. Nearly half (49.5%) of the patients were symptomatic at the time of admission, 28 patients (2.2%) developed symptoms post-testing during the course of illness, and 609 patients (48.5%) did not develop any symptom during the entire course of illness (Table 1). Most common presenting symptoms were fever (69.9%), cough (29.6%), generalized body pain (10.4%), difficulty in breathing (10.1%), fatigue (10.1%), anosmia (9.4%), and ageusia (8.9%) (Supplemental Table 1). Around 60.9% patients reported COVID-19 positivity among family members, and 649 (51.4%) were identified as a part of contact tracing. The majority of the patients (93.0%) had a normal neutrophil–lymphocyte ratio (NLR), and only 223 patients (17.7%) had any comorbidity (Table 1). Common comorbidities observed were diabetes mellitus (68.2%), systemic hypertension (31.4%), and coronary artery disease (6.3%) (Supplemental Table 2). Of the hypertensives, very few (1.1%) were on angiotensin-converting enzyme (ACE) inhibitors. Around 709 patients (60%) had taken the state government–recommended herbal decoction (Kabasura Kudineer/Nilavembu Kudineer) before the onset of illness and continued the herbal decoction till the duration of stay and 7 days post-discharge, and 200 (15.8%) patients had taken over-the-counter medications before admission to CCCs, the nature of which was not known. Smoking was reported by 120 (9.5%) patients and alcohol consumption by 160 (2.7%) patients. A majority (99.5%) did not have any international travel history, and 116 (9.2%) patients were part of a community gathering event in the national capital. The median duration between the date of isolation at CCC and date of discharge was 8 days (IQR: 6–10 days). The median duration between the date of isolation at CCC and date of referral was 3 days (IQR: 2–5 days).

**Table 1:** Basic characteristics of COVID-19 patients isolated at COVID Care Center, Chennai, Tamil Nadu, India

Characteristic	Number of COVID-19 cases (% of the total)
Age-group (years)	
0–18	149 (11.8)
19–45	807 (63.9)
46–60	263 (20.8)
Older than 60	44 (3.5)
Gender	
Male	838 (66.3)
Female	425 (33.7)
Presence of symptoms	
Symptomatic at the time of testing	626 (49.5)
Asymptomatic at the time of testing but developed symptoms during the course of illness	28 (2.2)
Asymptomatic throughout the course of illness	609 (48.2)
Neutrophil–lymphocyte ratio	
Less than 3.1	1,175 (93.0)
Greater than 3.1	88 (7.0)
Comorbidity	
Yes	223 (17.7)
No	1,040 (82.3)
Angiotensin-converting enzyme inhibitors	
Yes	13 (1.1)
No	1,250 (98.9)
Part of contact tracing	
Yes	649 (51.4)
No	597 (47.3)
Missing data	17 (1.3)
Any family member positive	
Yes	769 (60.9)
No	494 (39.1)
Smoking	
Yes	120 (9.5)
No	1,143 (90.5)
Alcohol	
Yes	160 (12.7)
No	1,103 (87.3)
Immune boosting	
Yes	749 (59.3)
No	514 (40.7)
Medicine taken before admission	
Yes	200 (15.8)
No	1,063 (84.2)
Travel history	
Yes	6 (0.5)
No	1,257 (99.5)
Community gathering	
Yes	116 (9.2)
No	1,147 (90.8)

On univariate analysis, it was found that symptomatic (OR: 13.60; 95% CI: 7.06–26.19) COVID-19 patients older than 50 years (OR: 2.82; 95% CI: 1.87–4.24) with comorbidities (OR: 10.85; 95% CI: 7.33–16.06) and a higher NLR (OR: 12.51; 95% CI: 7.81–20.04) were more likely to be referred because of the progression of illness (Table 2). COVID-19 patients who reported smoking (OR: 1.73; 95% CI: 1.02–2.93), alcohol consumption (OR: 2.17; 95% CI: 1.38–3.41), and to be on ACE inhibitors (OR: 5.58; 95% CI: 1.80–17.30) were also more likely to be referred because of disease progression (Table 2). COVID-19 patients identified and admitted to CCC as a part of contact tracing (OR: 0.36; 95% CI: 0.24–0.54) or with a history of family member being positive (OR: 0.54; 95% CI: 0.38–0.78) was less likely to progress to moderate/severe illness (Table 2). On multivariate analysis, symptomatic COVID-19 patients (adjusted OR [AOR]: 10.88; 95% CI: 5.08–23.30) with comorbidities (AOR: 8.40; 95% CI: 5.20–13.56) and higher NLR (AOR: 8.05; 95% CI: 4.51–14.36) were more likely to progress to severe illness and were referred to higher centers for treatment (Table 2).

**Table 2:** Factors associated with disease progression among asymptomatic and mildly symptomatic COVID-19 patients isolated in COVID Care Center, Chennai, Tamil Nadu, India

Characteristic	Number of patients with disease progression (% of the total) (*N* = 131)	Number of patients without disease progression (% of the total) (*N* = 1,132)	OR (95% CI)	*P*-value	Adjusted OR (95% CI)	*P*-value
Age-group (years)						
Older than 50	40 (30.5)	153 (13.51)	2.82 (1.87–4.24)	0.001	0.69 (0.38–1.21)	0.194
Younger than 50	91 (69.5)	979 (86.5)				
Gender						
Male	86 (65.65)	752 (66.44)	0.96 (0.66–1.41)	0.426	–	–
Female	45 (34.35)	380 (33.56)				
Presence of symptoms						
Symptomatic at the time of testing/presymptomatic	121 (92.34)	533 (47.08)	13.60 (7.06–26.19)	0.001	10.88 (5.08–23.30)	0.001
Asymptomatic throughout the course of illness	10 (7.66)	599 (52.92)				
Neutrophil–lymphocyte ratio						
Greater than 3.1	44 (33.58)	44 (3.9)	12.51 (7.81–20.04)	0.001	8.05 (4.51–14.36)	0.001
Less than 3.1	87 (66.42)	1,088 (96.1)				
Comorbidity						
Yes	80 (61.06)	143 (12.64)	10.85 (7.33–16.06)	0.001	8.40 (5.20–13.56)	0.001
No	51 (38.94)	989 (87.36)				
Angiotensin-converting enzyme inhibitors						
Yes	5 (3.81)	8 (0.70)	5.58 (1.80–17.30)	0.004	1.12 (0.29–4.27)	0.869
No	126 (96.19)	1,124 (99.30)				
Part of contact tracing						
Yes	38 (23.59)	611 (53.97)	0.36 (0.24–0.54)	0.001	1.40 (0.81–2.41)	0.229
No	87 (66.41)	509 (46.03)				
Any family member positive						
Yes	62 (47.33)	707 (62.46)	0.54 (0.38–0.78)	0.001	0.61 (0.38–0.98)	0.045
No	69 (52.67)	425 (37.54)				
Smoking						
Yes	19 (14.51)	101 (8.93)	1.73 (1.02–2.93)	0.025	1.32 (0.61–2.83)	0.476
No	112 (85.49)	1,031 (91.07)				
Alcohol						
Yes	29 (22.14)	131 (11.57)	2.17 (1.38–3.41)	0.001	1.31 (0.68–2.52)	0.417
No	102 (77.86)	1,001 (88.43)				
Immune boosting						
Yes	78 (59.55)	671 (59.27)	1.01 (0.70–1.46)	0.478	0.98 (0.62–1.56)	0.931
No	53 (40.45)	461 (40.73)				

OR = odds ratio.

## DISCUSSION

In our study, we have described demographic details, clinical characteristics, and outcomes of asymptomatic/mildly symptomatic COVID-19 patients isolated in a temporary makeshift-designated CCC in Chennai, Tamil Nadu, India. We also determined the risk factors for the progression of COVID-19 illness among the isolated patients warranting referral to a tertiary care center. Of the 198 patients who were transferred to a tertiary care hospital, 131 were transferred because of clinical progression of the disease, and others were transferred for reasons such as uncontrolled diabetes, hypertension, psychiatric illness with suicidal tendency, carpopedal spasm, and personal reasons. Regular patient monitoring identified nearly 131 (10.5%) isolated COVID-19 patients with disease progression who were promptly referred to higher center for advanced medical care. Fangcang shelter hospitals in China transferred around 13% of the isolated patients to a higher referral center, whereas community treatment centers (CTCs) in South Korea transferred only 2% of the isolated patients.^6,7^

Nearly half of the isolated COVID-19 patients in our study were asymptomatic. Half of the patients in our CCC were identified as a part of contact tracing, and two-thirds had a family member positive for COVID-19. Reasons for higher asymptomatic persons in our CCC may be due to extensive and meticulous tracing, and testing of close contacts thereby led to identification of more asymptomatic and presymptomatic cases. Proportion of asymptomatic cases in Fangcang shelter hospitals and CTCs in South Korea was 57.1% and 75.7%, respectively.^6,7^ Studies have documented asymptomatic patients contributing to the transmission of SARS-CoV-2 among the household members and community.^8,9^ Studies also recommend isolation of asymptomatic and mildly symptomatic COVID-19 patients to prevent further transmission.^10,11^ COVID Care Center plays a vital role in case isolation, thereby preventing transmission within households and community. Facilities in the CCC ensured monitoring of mildly symptomatic/asymptomatic patients who do not require hospitalized treatment. Establishment of such isolation centers made available hospital beds for moderate and severely ill patients.

Among the symptomatic patients, the most common presenting symptoms were fever, dry cough, myalgia or fatigue, dyspnea, and generalized body pain. Loss of smell and taste, symptoms touted to be more specific for COVID-19 during this pandemic, were found in approximately 10% of patients. The clinical symptoms were consistent with other studies reported globally.^12^ The patients were on enalapril and telmisartan, and the use of ACE inhibitors was identified as a risk factor in univariate analysis but not in the final model. Recent studies also reveal that individuals taking ACE inhibitors/receptors are not associated with increased risk of COVID-19 disease progression and mortality.^13^

Our study identified that symptomatic COVID-19 patients with comorbidities and a higher NLR isolated in CCC were more likely for clinical progression of the disease. Older patients and those with comorbidities were more prone to advance to severe disease and require referral to higher centers.^14,15^ Many studies have identified old age as a risk factor. But, in our study, old age was identified as a risk factor in univariate but was not significant in the final multivariate model. This might be due to lower proportion (6.5% above 60 years) of older age-group COVID-19 patients isolated in the CCC. More men were isolated in the CCC than women. But gender did not a play a role in disease progression among individuals isolated in the CCC, although findings from other studies suggest male gender as a risk factor for disease progression and mortality in COVID-19.^16^

Although only clinically stable patients were admitted in the CCC, a small percentage of patients clinically deteriorated during their stay. Identification of risk factors as to who clinically decline in a CCC is very important as focused attention can be given to these individuals. Symptomatic COVID-19 patients older than 60 years with comorbidities and a high NLR must be clinically monitored regularly.

We also found that 12% of referred patients did not show any clinical symptoms of disease progression, especially breathlessness, but on routine SpO_2_ monitoring, they were found to have low SpO_2_. We highly recommend that SpO_2_ be checked every 4 hours for these patients to promptly identify drop in saturation, provide oxygen therapy, and facilitate early referral to higher centers. COVID Care Center should be equipped with oxygen supply for providing oxygen support temporarily till patients get shifted to a tertiary care hospital.

Our study has three limitations. First, our study was a single-center study, and Chennai has around 28 such CCCs. Data from other centers could not be collected because of logistic reasons. However, our study CCC was one of the largest with 500 bedded capacity in Chennai city. Second, around 19% patients isolated at CCC could not be included in analysis because of incomplete data or failure to follow-up. Third, meticulous record-keeping was not possible during the initial stage of the epidemic, given the rapid surge of cases, and hence some of the information was collected during telephonic follow-up after discharge. Patients could have potentially concealed information, given the stigma associated with the disease.

## CONCLUSION

COVID Care Center isolation facilities are safe alternatives for medical institutions to isolate and monitor asymptomatic/mildly symptomatic COVID-19 patients. Proactive testing of close contacts of positive patients led to early diagnosis and isolation, thus preventing further transmission in the community. A majority of the patients isolated at CCC have an uneventful clinical course. Older symptomatic patients with comorbidities and a high NLR admitted in an isolation facility must be frequently monitored for prompt identification of clinical progression and referral to a higher center for advanced medical care.

## Supplemental tables

Supplemental materials
