# Supplementary material for: Clinical Outcomes among Asymptomatic or Mildly Symptomatic COVID-19 Patients in an Isolation Facility in Chennai, India
Source: Am J Trop Med Hyg. 2020 Nov 17;104(1):85–90. doi: 10.4269/ajtmh.20-1096 (PMC7790092; doi:10.4269/ajtmh.20-1096)
Supplement: Supplementary file 1 [file tpmd201096.SD1.pdf]

SUPPLEMENTAL TABLE 1. DETAILS OF SYMPTOMS AND ITS FREQUENCY AMONG SYMPTOMATIC PATIENTS BEFORE TESTING FOR COVID-19.

S. NO	SYMPTOMS	VALUE	PERCENTAGE
**1.**	FEVER	438	69.97%
**2.**	COUGH DRY	185	29.55%
**3.**	GENERALISED BODY PAIN	65	10.38%
**4.**	BRATHLESSNESS	63	10.06%
**5.**	FATIGUE	63	10.06%
**6.**	ANOSMIA	59	9.42%
**7.**	AGEUSIA	56	8.95%
**8.**	SNEEZING AND RUNNING NOSE	54	8.63%
**9.**	SORE THROAT	44	7.03%
**10.**	HEAD ACHE	36	5.75%
**11.**	PRODUCTIVE COUGH	22	3.51%
**12.**	DIARHOEA	20	3.19%
**13.**	VOMITING	15	2.40%
**14.**	JOINT PAIN	10	1.60%
**15.**	CHEST PAIN NON-SPECIFIC	9	1.44%
**16.**	SEVERE BACK PAIN	8	1.28%
**17.**	ABDOMINAL PAIN	4	0.64%
**18.**	GIDDINESS	4	0.64%
**19.**	ANOREXIA	3	0.48%
**20.**	WHEEZING	3	0.48%

**21.**	HEMOPTYSIS	2	0.32%
**22.**	MAYALGIA	2	0.32%
**23.**	ANGINAL CHEST PAIN	1	0.16%
**24.**	ITCHING AND DISCHARGE FROM EYES	1	0.16%
**25.**	RASHES	1	0.16%

SUPPLEMENTAL TABLE 2. DETAILS FOR CO-MORBIDITY AND ITS FREQUENCY AMONG COVID -19 PATIENTS.

S. NO	CO-MORBIDITY AMONG COVID-19 PATIENTS	FREQUENCY AMONG TOTAL COMORBIDITY (N = 223)	PERCENTAGE
**1.**	DIABETES MELLITUS	152	68.16%
**2.**	SYSTEMIC ARTERIAL HYPERTENSION	70	31.39%
**3.**	CORONARY ARTERY DISEASE	14	6.28%
**4.**	HYPOTHYROID	13	5.83%
**5.**	BRONCHIAL ASTHMA	10	4.48%
**6.**	CHRONIC OBSTRUCTIVE PULMONARY DISEASE	8	3.59%
**7.**	CHRONIC KIDNEY DISEASE	7	3.14%
**8.**	ASTHMA	3	1.35%
**9.**	ISCHEMIC HEART DISEASE	3	1.35%
**10.**	SEIZURE	3	1.35%
**11.**	CHRONIC NEUROLOGICAL DISORDER	2	0.90%
**12.**	RHEUMATOID ARTHRITIS	2	0.90%
**13.**	STONES	2	0.90%
**14.**	STROKE	2	0.90%

**15.**	ALCOHOLIC FATTY LIVER DISEASE	1	0.45%
**16.**	ANGINA	1	0.45%
**17.**	BENIGN PROSTATIC HYPERPLASIA	1	0.45%
**18.**	CANCER	1	0.45%
**19.**	CKD ON MHD HCV/HINC	1	0.45%
**20.**	DIABETES MELLITUS INSULIN	1	0.45%
**21.**	DIABETES MELLITUSUNCONTROLLED	1	0.45%
**22.**	HEMIPLEGIA	1	0.45%
**23.**	HERNIA	1	0.45%
**24.**	HIV ON ART	1	0.45%
**25.**	ORTHOPNEA	1	0.45%
**26.**	PERIPHERAL VASCULAR DISEASE	1	0.45%
**27.**	RHEUMATIC HEART DISEASE	1	0.45%
**28.**	CRANIOTOMY	1	0.45%
**29.**	TUBERCULOSIS	1	0.45%

SUPPLEMENTAL TABLE 3. CHARACTERISTICS OF PATIENTS WHO DIED OF CORONAVIRUS DISEASE 2019

	PATIENT 1	PATIENT 2	PATIENT 3
Age	68	56	60
Sex	Male	Female	Female
Spo2 saturation at the time of referral	79%	85%	85%
Mean NLR	3.9	3.6	5.4
Co-morbidity	Diabetes Mellitus, Systemic Hyper Tension	Stroke	Systemic Hyper Tension, Rheumatoid Arthritis)
Symptomatic	Fever, Cough	Fever, Cough, Breathlessness	Cough, Breathlessness
Part of Contact Tracing	No	No	No
Family Positivity	No	Yes	No
Smoking	No	No	No
Alcohol	No	No	No
